# Efficacy and Safety of Complementary Therapy With Jing Si Herbal Tea in Patients With Mild-To-Moderate COVID-19: A Prospective Cohort Study

**DOI:** 10.3389/fnut.2022.832321

**Published:** 2022-03-14

**Authors:** Po-Chun Hsieh, You-Chen Chao, Kuo-Wang Tsai, Chung-Hsien Li, I-Shiang Tzeng, Yao-Kuang Wu, Cheng Yen Shih

**Affiliations:** ^1^Department of Chinese Medicine, Taipei Tzu Chi Hospital, Buddhist Tzu Chi Medical Foundation, New Taipei City, Taiwan; ^2^Department of Internal Medicine, Taipei Tzu Chi Hospital, Buddhist Tzu Chi Medical Foundation, New Taipei City, Taiwan; ^3^School of Medicine, Tzu Chi University, Hualien, Taiwan; ^4^Department of Research, Taipei Tzu Chi Hospital, Buddhist Tzu Chi Medical Foundation, New Taipei City, Taiwan; ^5^Division of Pulmonary Medicine, Department of Internal Medicine, Taipei Tzu Chi Hospital, Buddhist Tzu Chi Medical Foundation, New Taipei City, Taiwan; ^6^Buddhist Tzu Chi Medical Foundation, Hualien, Taiwan

**Keywords:** mild-to-moderate COVID-19, Jing Si Herbal Tea, complementary and alternative medicine, traditional Chinese medicine, phytomedicine, herbal medicine

## Abstract

**Background:**

Since late 2019, there has been a global COVID-19 pandemic. To preserve medical capacity and decrease adverse health effects, preventing the progression of COVID-19 to severe status is essential. Jing-Si Herbal Tea (JSHT), a novel traditional Chinese medicine formula was developed to treat COVID-19. This study examined the clinical efficacy and safety of JSHT in patients with mild-to-moderate COVID-19.

**Methods:**

In this prospective cohort study, we enrolled 260 patients with mild-to-moderate COVID-19. The enrolled patients were divided into the JSHT (*n* = 117) and control (*n* = 143) groups. Both groups received standard management. The JSHT group was treated with JSHT as a complementary therapy.

**Results:**

Compared with standard management alone, JSHT combined with standard management more effectively improved the reverse transcription–polymerase chain reaction cycle threshold value, C-reactive protein level, and Brixia score in the adult patients with mild-to-moderate COVID-19, especially in the male and older patients (those aged ≥60 years). The results revealed that the patients treated with JSHT combined with standard management had 51, 70, and 100% lower risks of intubation, Medisave Care Unit admission, and mortality compared with those receiving standard management only.

**Conclusions:**

JSHT combined with standard management more effectively reduced the SARS-CoV-2 viral load and systemic inflammation and alleviated lung infiltrates in the patients with mild-to-moderate COVID-19, especially in the male and older patients (those aged ≥60 years). JSHT combined with standard management may prevent critical status and mortality in patients with mild-to-moderate COVID-19. JSHT is a promising complementary therapy for patients with mild-to-moderate COVID-19.

## Introduction

In late 2019, multiple cases of novel viral pneumonia were reported in Wuhan City, China. Since then, the SARS-CoV-2 virus has spread rapidly, leading to the global COVID-19 pandemic (1), which has considerably affected patient health and health-care systems (2). SARS-CoV-2 is primarily transmitted through direct or indirect respiratory tract exposure (3). After binding to the angiotensin-converting enzyme 2 (ACE2) receptor on the respiratory epithelium, SARS-CoV-2 begins replicating, migrates down to the airway, and enters alveolar epithelial cells in the lungs, thus resulting in asymptomatic infection progressing to severe respiratory failure (3). Most unvaccinated patients develop asymptomatic, mild, or moderate COVID-19 infection. However, in some patients, epithelial cells in the respiratory tract stimulate immune cells, resulting in a cytokine storm, which is considered the leading cause of the severe clinical manifestation and mortality in patients with COVID-19 (4). To preserve medical capacity and reduce adverse health effects on individuals, preventing the progression of asymptomatic, mild, or moderate COVID-19 infection into severe COVID-19 is essential.

In January 2022, five SARS-CoV-2 variants have been designated as variants of concern (VOC) by the World Health Organization: Alpha, Beta, Gamma, Delta, and Omicron (5). The variants have been spread worldwide, and the dominant VOC have changed over time (5). Various possible mutation pathways of SARS-CoV-2 have been proposed, including ligand-receptor interactions, genomic alterations, lysosomal activity, recombination, conditional mutations, complementation, genetic robustness, and benign relationship (6). Global pandemic outbreaks and genome sequence profiles revealed that SARS-CoV-2 has a high mutation potential and high infectivity, enabling the virus to evolve resistance to newly developed therapeutic strategies rapidly (6). As the pandemic progressed, the mutation of the SARS-CoV-2 genome led to different clinical and socio-economical impacts, which observed dynamic risks of transmissibility, vaccine breakthrough infection, hospitalization, and critical condition (5).

Many COVID-19 vaccines have been proven to prevent hospitalization and mortality in adults (7). To date (January 19, 2022), 60.1% of the world population has received at least one dose of a COVID-19 vaccine. However, only 9.6% of people in low-income countries have received at least one dose (8). The BNT162b2 vaccine in 12-to-15-year-old recipients has been reported favorable safety and highly effective against COVID-19 (9). However, due to the limited information from short-term trials and high post-inoculation severe adverse effects and deaths, there are still concerns that lag the vaccination rate on children/adolescents (10).

In 2020, compared with most industrialized countries, Taiwan was not considerably affected by the COVID-19 pandemic, which can be attributed to rapid national border control, nonpharmaceutical interventions, and cooperation between the government and people (11, 12). However, an outbreak of the Alpha variant of SARS-CoV-2 occurred in mid-May 2021, at which point a relatively low proportion of individuals had received the vaccine (0.55 doses administered per 100 people) (13). Except for standard management, potential complementary therapies for preventing severe COVID-19 should be investigated.

Emerging evidence indicates that the combination of traditional Chinese medicine (TCM) and standard management may play a vital role in treating patients with COVID-19 (14–17). The findings of *in vitro* pharmacological assays demonstrated the efficacy of Taiwan Chingguan Yihau (NRICM101) in inhibiting the spike protein/ACE2 interaction, 3CL protease activity, viral plaque formation, and cytokine production (e.g., production of interleukin [IL]-6 and tumor necrosis factor [TNF]-α) (14). Because of its antiviral and anti-inflammatory effects, NRICM101 may be used to inhibit SARS-CoV-2 invasion and proliferation (14); however, the changes that can be achieved in clinical parameters and outcomes remain unclear. During the pandemic, the Chinese government recommended 6 TCM formulas, referred to as “3-drugs-3-formulas,” for treating SARS-CoV-2 infection (18). Five of the formulas were based on the core Maxing Shigan decoction, which possesses anti-inflammatory and antiviral properties (18). Studies have reported that TCM as an adjuvant therapy combined with conventional treatment may be effective and safe for treating mild-to-moderate COVID-19 (15–17). However, because most of the results have been reported as odds ratios, including the improvement rate of symptoms or lung images, quantification of the clinical effects is difficult.

Jing Si Herbal Tea (JSHT), a novel TCM formula, was developed to treat COVID-19. The ingredients of JSHT have been reported to exert anti-SARS-CoV-2, anti-inflammatory, and antithrombotic effects, thus targeting the main pathophysiological pathways in COVID-19 (14, 19–23). We hypothesized that JSHT effectively shortens the viral shedding duration and alleviates respiratory and systemic inflammation. This study analyzed the clinical efficacy and safety of JSHT in patients with mild-to-moderate COVID-19.

## Materials and Methods

### Study Design and Participants

In this prospective cohort study, we enrolled patients with mild-to-moderate COVID-19 upon admission (immediately after the confirmation of COVID-19 infection) from one medical center in New Taipei City between May 1 and August 31, 2021. The study protocol and informed consent form were approved by the Research Ethics Committee of Taipei Tzu Chi Hospital, Buddhist Tzu Chi Medical Foundation (No. 10-X-045). All participants provided written informed consent.

The inclusion criteria were as follows: (1) laboratory-confirmed positive COVID-19 based on reverse transcription–polymerase chain reaction (RT-PCR) testing, (2) age ≥ 18 years, and (3) mild-to-moderate COVID-19 (24). The exclusion criteria were as follows: (1) critical status requiring mechanical ventilation; (2) having severe systemic disease (i.e., malignancy or autoimmune, liver, or renal diseases); (3) pregnancy or lactation in women; (4) participation in other clinical trials within 3 months; (5) history of allergy to the investigational medications; and (6) other conditions judged by the investigators.

The enrolled patients were divided into the JSHT and control groups. Patients in both groups were treated with standard management—supportive treatment, oxygen therapy, symptomatic therapies, and COVID-19-specific medications—in accordance with the Interim Guidelines for Clinical Management of SARS-CoV-2 Infection (11th edition, 2021) (25). Patients in the JSHT group were treated with JSHT as a complementary therapy.

The patients' baseline demographic and clinical characteristics were collected before the research program upon admission (on day 1 [D1]). The following variables were recorded: sex, age, body mass index (BMI), smoking status, hemogram value, serum biochemistry profile, and Charlson comorbidity index. The key clinical parameters: peripheral oxygen saturation (SpO2), fraction of inspired oxygen (FiO2), neutrophil-to-lymphocyte ratio (NLR), RT-PCR cycle threshold (CT) value, C-reactive protein (CRP) level, and Brixia score (26), and serum cytokine levels (IL-6, IL-8, and IL-10) levels were also examined and recorded. In addition, the key clinical parameters, liver and renal function parameters, and cytokine levels were evaluated and recorded after the research program (on day 8 [D8]). The use of concomitant medications (antibiotics, dexamethasone, remdesivir, and tocilizumab), clinical outcomes (including intubation, Medisave Care Unit [MICU] admission, discharge, and mortality), and adverse events during and after the research program were also recorded.

### Jing Si Herbal Tea

JSHT is a novel TCM herbal formula developed by the Buddhist Tzu Chi Medical Foundation, Taiwan. The ingredients of JSHT include Houttuyniae Herba (Hanyu Pinyin: Yu Xing Cao; scientific name: *Houttuynia cordata* Thunb.; HC; percentage by weight: 14.18%), Perillae Folium (Zi Su Ye; *Perilla frutescens*; PF; 7.09%), Glycyrrhizae Radix et Rhizoma (Gan Cao; *Glycyrrhiza glabra*; GG; 7.09%), Artemisiae Argyi Folium (Ai Ye; *Artemisia argyi*; AA; 21.28%), *Anisomeles indica* (L.) Kuntze (Yu Zhen Cao; AI; 21.28%), Platycodonis Radix (Jie Geng; *Platycodon grandiflorus*; PG; 14.18%), Ophiopogonis Radix (Mai Men Dong; *Ophiopogon japonicus*; OJ; 14.18%), and Chrysanthemi Flos (Ju Hua; *Chrysanthemum morifolium*; CM; 0.71%). Aqueous extraction of the herbs was filtered and concentrated to obtain a JSHT potion. The JSHT used in the current study was a standardized manufactured product with vacuum packaging that contained a single-dose (225 mL) potion. The JSHT group were administered a single-dose potion 3 times daily for 7 days.

### Evaluation of IL-6, IL-8, and IL-10 Concentrations by Using Enzyme-Linked Immunosorbent Assay

We evaluated the concentrations of IL-6, IL-8, and IL-10 in the serum of patients with COVID-19 by using the following enzyme-linked immunosorbent assay (ELISA) kits: IL-6 (88-7066, Thermo Fisher Scientific Inc., Waltham, MA, USA), IL-8 (88-8086, Thermo Fisher Scientific Inc.), and IL-10 (88-7106, Thermo Fisher Scientific Inc.). After performing ELISA in accordance with the manufacturer's instructions, absorbance was read at 450 nm on a Tecan infinite M200 PRO reader (Tecan, Maennedorf, Switzerland).

### Statistical Analysis

All statistical analyses were conducted using GraphPad Prism 9 for macOS (Version 9.2.0, GraphPad Software, San Diego, CA, USA, www.graphpad.com). The baseline demographic and clinical characteristics are presented as the patient number (%) and mean ± standard deviation (SD). Outcome measurements are presented as the mean ± SD. For intragroup and intergroup comparisons, categorical variables were examined using Fisher's exact test and the chi-square test, whereas continuous variables were examined using the independent *t*-test, Wilcoxon signed-rank test, and Mann–Whitney U test. Analysis of covariance (ANCOVA) for statistical control was performed using software R 4.1.1 for Windows (27) in comparisons of changes in clinical parameters with baseline intragroup difference. A 2-tailed *P* value of < 0.05 was considered statistically significant.

## Results

### Cohort Characteristics

**Figure 1:**
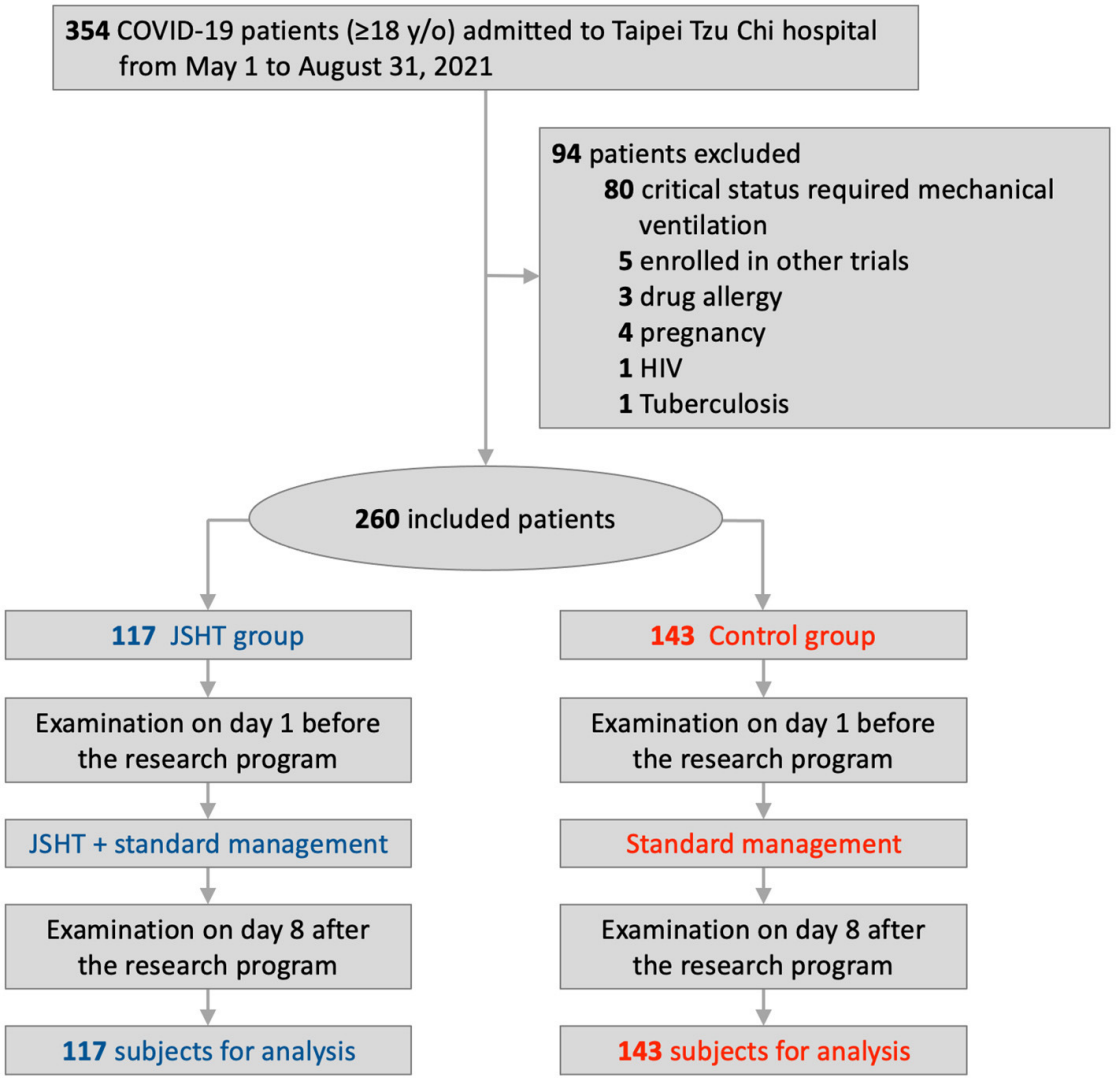
Study flow diagram.

Figure 1 presents the study flow diagram. A total of 354 patients with mild-to-moderate COVID-19 infection were admitted to Taipei Tzu Chi Hospital between May 1 and August 31, 2021. After applying the exclusion criteria, 94 patients were excluded (68 patients who were intubated within 1 week or admitted to the MICU immediately after hospitalization, 12 patients who had impending respiratory failure and a do-not-resuscitate order, 5 patients who were enrolled in other trials, 3 patients who had drug allergies, 4 patients who were pregnant, 1 patient who had human immunodeficiency virus infection, and 1 patient who had tuberculosis infection). Overall, 260 patients were included in this study and divided into the JSHT group (117 patients) and the control group (143 patients).

Table 1 lists the baseline demographic and clinical characteristics of the patients. No significant differences in sex, age, BMI, smoking status, hemogram value, serum biochemistry profile, or Charlson comorbidity index were observed between the JSHT and control groups. The mean age and BMI of the 117 participants in the JSHT group were 55.42 ± 15.38 (range, 20–95) years and 24.59 ± 15.38 (range, 17.48–32.89) kg/m^2^, respectively. The mean age and BMI of the 143 participants in the control group were 53.96 ± 16.97 (range, 19–97) years and 25.58 ± 4.66 (range, 16.11–44.58) kg/m^2^, respectively.

**Table 1:** Baseline demographics and clinical characteristics upon admission.

**Variable**	**JSHT group**	**Control group**	***p* value**
Number of patients, n	117	143	
Male, *n* (%)	56 (47.9)	70 (49.0)	0.960
Female, *n* (%)	61 (25.1)	73 (51.0)	
Age, year, mean ± SD	55.42 ± 15.38	53.96 ± 16.97	0.472
BMI, kg/m^2^, mean ± SD	24.59 ± 3.16	25.58 ± 4.66	0.056
Smoking status, n (%)			0.254
Never-smoker	87 (74.4)	117 (81.8)	
Current smoker	17 (14.5)	12 (8.4)	
Ex-smoker	13 (11.1)	14 (9.8)	
Hemogram value, mean ± SD			
Leukocyte count, 10^9^/L	5,257.09 ± 1,758.39	5,496.63 ± 2,230.94	0.345
Neutrophil count, %	68.59 ± 11.28	70.37 ± 11.31	0.208
Lymphocyte count, %	22.86 ± 10.22	20.85 ± 9.62	0.105
Hemoglobin, g/dL	13.49 ± 1.86	13.62 ± 2.13	0.597
Platelet count, 10^3^ μL	198.09 ± 64.06	194.66 ± 77.65	0.702
Serum biochemistry profile, mean ± SD			
AST, U/L	33.15 ± 20.82	37.84 ± 28.10	0.165
ALT, U/L	33.78 ± 33.07	32.15 ± 31.08	0.690
Total bilirubin, mg/dL	0.58 ± 0.21	0.62 ± 0.41	0.395
BUN, mg/dL	15.52 ± 22.06	14.90 ± 10.62	0.772
Creatinine, mg/dL	0.83 ± 0.41	1.14 ± 2.08	0.088
D-dimer	755.36 ± 880.67	1,005.28 ± 1,330.51	0.132
Ferritin	570.56 ± 695.06	682.13 ± 1,296.09	0.514
Charlson comorbidity index, mean ± SD	1.75 ± 1.73	1.75 ± 1.93	0.987
Key clinical paremeters, mean ± SD			
SpO_2_, %	95.86 ± 1.86	95.96 ± 2.43	0.323
FiO_2_, %	23.28 ± 11.03	22.72 ± 9.63	0.659
Neutrophil-to-lymphocyte ratio	4.52 ± 5.73	4.93 ± 4.63	0.152
RT-PCR CT value	21.21 ± 6.10	23.38 ± 6.62	0.006*
CRP, mg/dL	4.33 ± 5.00	3.89 ± 4.33	0.450
Brixia score	2.03 ± 2.22	1.27 ± 1.61	0.003*

ALT, alanine aminotransferase; AST, aspartate aminotransferase; BMI, body mass index; BUN, blood urea nitrogen; CRP, c-reactive protein; FiO_2_, fraction of inspired oxygen; JSHT, Jing Si Herbal Tea; RT-PCR CT value, reverse transcription polymerase chain reaction cycle threshold value; SpO_2_, peripheral oxygen saturation. ^*^Indicates statistical significance (p < 0.05).

Regarding the key clinical parameters, no significant differences in SpO2, FiO2, NLR, or CRP were observed between the JSHT and control groups. However, the mean RT-PCR CT value was significantly lower (*P* =0.006) in the JSHT group (21.21 ± 6.10; range 9–34) than in the control group (23.38 ± 6.62; range 11–38). The mean Brixia score was significantly higher (*P* = 0.003) in the JSHT group (2.03 ± 2.22; range 0–10) than in the control group (1.27 ± 1.61; range 0–7). The JSHT group had higher viral load and more severe lung infiltrates at admission than did the control group.

### Management During the Study

The patients in both the groups received standard management. The use of concomitant medications (antibiotics, dexamethasone, remdesivir, and tocilizumab) and oxygen therapy with high-flow nasal cannula (HFNC) was recorded. No significant differences in the use of concomitant medications or oxygen therapy with HFNC were noted between the JSHT and control groups (Supplementary Table S1).

### Clinical Effects of Complementary Therapy With JSHT

**Figure 2:**
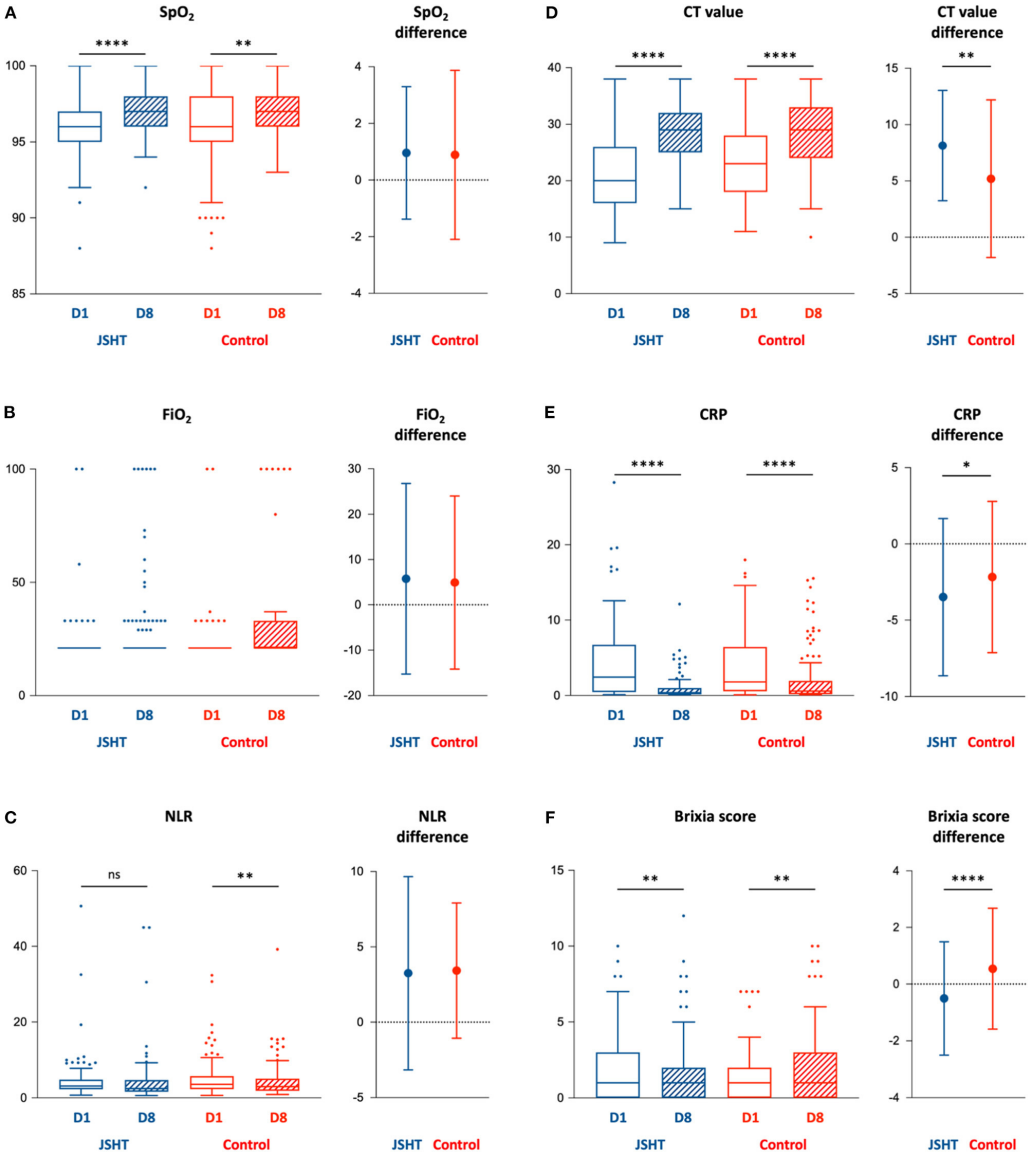
Key parameters before (D1) and after (D8) the study. **(A)** SpO_2_; **(B)** FiO_2_; **(C)** NLR; **(D)** CT value; **(E)** CRP; **(F)** Brixia score. BMI, body mass index; CRP, c-reactive protein; CT value, Reverse Transcription Polymerase Chain Reaction cycle threshold value; FiO2, fraction of inspired oxygen; JSHT, Jing Si Herbal Tea; NLR, Neutrophil-to-lymphocyte ratio; SpO_2_, peripheral oxygen saturation. *p < 0.05, **p < 0.01, ****p < 0.0001.

The clinical effects of complementary therapy with JSHT on the patients with mild-to-moderate COVID-19 infection are presented in Figure 2 and Supplementary Table S2. The results demonstrated that standard management resulted in significantly improved SpO2, NLR, CT value, CRP level, and Brixia score after the study (on D8 compared with D1) in the control group. JSHT combined with standard management resulted in significantly improved SpO2, CT value, CRP level, and Brixia score after the study (on D8 compared with D1). The increase of CT value after the study was significantly higher (*P* = 0.001) in the JSHT group (8.14 ± 4.90) than in the control group (5.20 ± 6.99; Figure 2D). The decrease of CRP after the study was significantly greater (*P* =0.044) in the JSHT group (−3.48 ± 5.15 mg/dL) than in the control group (−2.17 ± 4.96 mg/dL; Figure 2E). The decrease of Brixia score after the study was significantly greater (*P* < 0.0001) in the JSHT group (−0.50 ± 1.99) than in the control group (0.55 ± 2.14; Figure 2F).

Concerning the intragroup differences of baseline CT value and Brixia score between the JSHT and control groups, we conducted ANCOVA using the baseline values as covariates to further evaluate the results. The ANCOVA results revealed that after the study, the CT value and Brixia score were more significantly improved in the JSHT group (*P* < 0.001 and *P* < 0.0001, respectively). JSHT combined with standard management more effectively reduced the SARS-CoV-2 viral load and systemic inflammation and alleviated lung infiltrates in the patients with mild-to-moderate COVID-19 infection.

### Subgroup Analysis of the Clinical Effects of Complementary Therapy With JSHT

**Figure 3:**
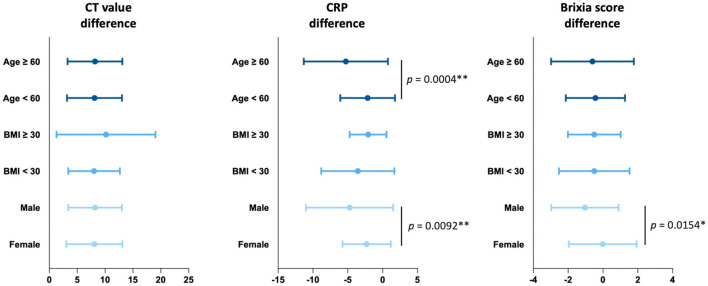
Subgroup analysis of the effects of JSHT on differences in the CT value, CRP level, and Brixia score. CRP, c-reactive protein; CT value, Reverse Transcription Polymerase Chain Reaction cycle threshold value. *p < 0.05, **p < 0.01.

We conducted subgroup analysis to investigate the clinical effects of complementary therapy with JSHT under different risk factors, including age (≥60 or <60 years), (28) BMI (≥30 or <30), (29) and sex (male or female) (30) (Figure 3). The results demonstrated that JSHT combined with standard management reduced the SARS-CoV-2 viral load in all subgroups. Furthermore, JSHT combined with standard management significantly and more effectively reduced systemic inflammation in the patients aged ≥60 years (*P* = 0.0004) and male patients (*P* = 0.0092). In addition, JSHT combined with standard management significantly and more effectively alleviated lung infiltrates in the male patients (*P* = 0.0154). In summary, JSHT was observed to be suitable for all the groups of adult patients, especially male and older patients (those aged ≥60 years).

### Effects of Complementary Therapy With JSHT on Cytokine Levels

The baseline IL-6 levels were 8.69 ± 17.79 pg/mL and 11.45 ± 21.78 pg/mL in the JSHT and control groups, respectively (*P* = 0.549), whereas the corresponding baseline IL-8 levels were 9.73 ± 9.43 pg/mL and 11.83 ± 10.36 pg/mL (*P* = 0.368). The baseline IL-10 levels were 10.10 ± 9.07 pg/mL and 9.33 ± 7.70 pg/mL in the JSHT and control groups, respectively (*P* = 0.842). No significant differences in IL-6, IL-8, or IL-10 level were noted between the JSHT and control groups. The IL-6, IL-8, and IL-10 levels were significantly lower after the study in both groups. However, the changes in these levels did not significantly differ between the groups (Supplementary Table S3).

### Effects of Complementary Therapy With JSHT on Clinical Outcomes

Details regarding the effects of complementary therapy with JSHT on clinical outcomes are presented in Table 2. The incidence of intubation after the study was 1.7% in the JSHT group and 3.5% in the control group. The relative risk was 0.49 (95% CI: 0.11–2.12, *P* = 0.6102). The incidence of MICU admission after the study was 3.4% in the JSHT group and 4.9% in the control group. The relative risk was 0.70 (95% CI: 0.22–2.18, *P* = 0.7805). The incidence of mortality after the study was 0.0% in the JSHT group and 2.8% in the control group. The relative risk was 0.00 (95% CI: 0.00–1.16, *P* = 0.1879). The results suggested that the patients with mild-to-moderate COVID-19 infection treated with JSHT combined with standard management had 51%, 30%, and 100% lower risks of intubation, MICU admission, and mortality, respectively, compared with the patients treated with standard management only. Although no significant differences were discovered between the groups, JSHT combined with standard treatment tended to prevent critical status and mortality in the patients with mild-to-moderate COVID-19 infection. To validate the results, double-blinded, prospective, randomized controlled trials using a larger population are warranted in the future.

**Table 2:** Clinical outcomes after the study.

**Variable**	**JSHT group**	**Control group**	**Relative risk JSHT/Control**	***p* value**	**95% CI**
Critical status, *n* (%)					
Intubation	2 (1.7)	5 (3.5)	0.49	0.6102	0.11 to 2.12
MICU admission	4 (3.4)	7 (4.9)	0.70	0.7805	0.22 to 2.18
Clinical outcome, *n* (%)					
Discharge	117 (100)	139 (97.2)	1.03	0.1879	0.99 to 1.04
Mortality	0 (0)	4 (2.8)	0.00	0.1879	0.00 to 1.16

*JSHT, Jing Si Herbal Tea; MICU, Medisave Intensive Care Unit*.

### Safety of Complementary Therapy With JSHT

We examined adverse effects, liver function, and renal function to evaluate the safety of complementary therapy with JSHT. Only 4 patients developed diarrhea after receiving JSHT, and their symptoms disappeared spontaneously within 3 days. No other adverse effects were observed. The liver and renal function values (aspartate aminotransferase, alanine aminotransferase, total bilirubin, blood urea nitrogen, and creatinine) were within normal ranges in both the JSHT and control groups before and after the study. The results indicate that complementary therapy with JSHT is safe for patients with mild-to-moderate COVID-19 infection (Supplementary Table S4).

## Discussion

This is the first study to investigate the efficacy and safety of JSHT combined with standard management in patients with mild-to-moderate COVID-19. The results demonstrated that JSHT combined with standard management more effectively reduced the SARS-CoV-2 viral load and systemic inflammation and alleviated lung infiltrates in adult patients with mild-to-moderate COVID-19, especially in male and older patients (those aged ≥60 years). In addition, the patients treated with JSHT combined with standard management had 51, 30, and 100% lower risks of intubation, MICU admission, and mortality compared with the patients treated with standard management only. This finding indicates the potential of complementary JSHT treatment in preventing critical status and mortality.

The hyperinflammatory response induced by SARS-CoV-2 is a major cause of disease severity and death in COVID-19 (31). Increased IL-6 level is associated with COVID-19 severity and poor prognosis (32). Zhang et al. reported that an IL-6 concentration of >37.65 pg/mL was predictive of in-hospital death (33). Our findings revealed low IL-6 levels in both the JSHT and control groups before and after the study, indicating that the included patients with COVID-19 had relatively low disease severity and low mortality risk. IL-8 is a proinflammatory cytokine that may recruit neutrophils to the infected areas and is associated with tissue damage (34). Li et al. demonstrated that IL-8 is a sensitive biomarker in patients with mild or severe COVID-19, whereas IL-6 is a biomarker of severe COVID-19 (34). Ma et al. reported that patients with COVID-19 with a high IL-8 level (≥10.65 pg/mL) had a significantly longer illness duration than did those with a low IL-8 level (<10.65 pg/mL) (35). We observed low serum IL-8 levels in both the JSHT and control groups after the study. IL-10 has potent anti-inflammatory and immunosuppressive effects (31). An increase in the IL-10 level can be interpreted as an attempt to suppress hyperinflammation (36). However, a marked early increase in the level of the proinflammatory cytokine IL-10 may be associated with COVID-19 severity (37). Han et al. reported that IL-6 and IL-10 are disease severity predictors of COVID-19 (38). Our results revealed low serum IL-10 levels in both the JSHT and control groups after the study. Although CRP levels were more significantly improved in the JSHT group compared with the control group, no significant differences in changes in IL-6, IL-8, or IL-10 levels were observed between the groups. The multiple active ingredients of JSHT may exert an anti-inflammatory effect through multiple signaling pathways and thus contribute to reducing systemic inflammation.

JSHT potentially targeted 3 main pathophysiological pathways: anti-infective, anti-inflammation, and anti-thrombosis. HC, GG and PF exert an anti-infective effect against SARS-CoV-2 (14, 19, 39–41). The main protease (M^pro^) of SARS-CoV-2 is a crucial enzyme of coronaviruses and plays a pivotal role in mediating viral replication and transcription (42). HC blocks binding between ACE2 and the spike protein of SARS-CoV-2 (14). Regarding the active ingredients of HC, 6-hydroxyondansetron has higher binding affinity toward 2 SARS-CoV-2 receptor proteins, namely M^pro^ and papain-like protease (PL^pro^). In addition, quercitrin has been identified as another promising inhibitor because it exhibits the highest binding affinity toward the ADP ribose phosphatase of SARS-CoV-2 (19, 39). GG exerts an anti-infective effect (19). The main active ingredients of GG, glycyrrhizin, targeted the ACE2 receptor with structural affinity and prevented SARS-CoV-2 entry in silicon and docking studies (40, 41). Moreover, PF exerts an anti-infective effect and significantly reduced the pulmonary viral load (*in vivo* anti-SARS-CoV-2 assay) in a female golden Syrian hamster model (19).

HC, AA, AI, and PG exerts anti-inflammatory effects against pulmonary inflammation. The phytoconstituents of HC, including afzelin, hyperoside and, quercitrin, could reduce inflammation (43). HC extracts and the bioactive molecules in HC possess both anti-inflammatory and anti-oxidative properties (43). AA and its active compound, dehydromatricarin A, markedly reduced pulmonary inflammation by suppressing inducible nitric oxide synthase (iNOS) expression and nuclear factor kappa B (NF-κB) phosphorylation, thus reducing the levels of TNF-α and IL-6 (20). AI inhibited the inflammatory mediator nitric oxide and TNF-α and IL-12 production in lipopolysaccharide/IFN-γ-activated macrophages (21). PG was discovered to exert apophlegmatic, antitussive, anti-inflammatory, and antioxidative effects (22).

Thrombotic events that occur in COVID-19 are strongly associated with increased disease severity and poor clinical outcomes (44). Distinctive microvascular abnormalities in COVID-19 include endothelial inflammation, intercellular junction disruption, and microthrombus formation (44). OJ exerted antithrombotic effects by inhibiting venous thrombosis mainly through protecting endothelial cells and reducing leukocyte–endothelial cell adhesion (23).

Based on the *in vivo* and *in vitro* experiments, NRICM101 inhibits SARS-CoV-2 invasion and proliferation through its antiviral and anti-inflammatory properties (14). In clinical practice, NRICM101 was used as an optional alternative therapy in treating COVID-19 in Taiwan and was observed to be effective. However, statistically analyzed real-world data have not been reported. According to previous meta-analysis studies, the combination of traditional Chinese herbal medicine with conventional therapy was effective and safe in the treatment of mild to moderate COVID-19, which improved lung CT parameters, CRP, and clinical symptoms (fever, cough, and fatigue) (15–17). The formulas showed similar therapeutical effects as JSHT (15–17). According to the preliminary data, the worldwide dominant Omicron variant shows increased transmissibility compared to Delta and may evade vaccine-induced immunity (45). As more patients may quarantine at home, JSHT, suitable for mild-to-moderate COVID-19, is also suggested for home care management.

### Limitation of the Study

This study has some limitations that should be addressed. First, this is not a double-blinded, randomized controlled trial. Second, the dosage and duration of JSHT treatment were established empirically. Studies with more groups and different dosages and durations are warranted. Third, the molecular mechanisms through which JSHT combats COVID-19 remain unclear. Double-blinded, prospective, randomized controlled trials with a larger population and basic studies investigating the underlying mechanisms should be conducted to comprehensively investigate the effects of JSHT.

## Conclusions

JSHT combined with standard management more effectively reduced the SARS-CoV-2 viral load and systemic inflammation and alleviated lung infiltrates in patients with mild-to-moderate COVID-19, especially in male and older patients (those aged ≥60 years). JSHT combined with standard management may prevent critical status and mortality in patients with mild-to-moderate COVID-19. Concerning the efficacy and safety of JSHT, we suggest JSHT as a promising complementary therapy for patients with mild-to-moderate COVID-19.

## Data Availability Statement

The original contributions presented in the study are included in the article/Supplementary Material, further inquiries can be directed to the corresponding author.

## Ethics Statement

The studies involving human participants were reviewed and approved by Research Ethics Committee of Taipei Tzu Chi Hospital, Buddhist Tzu Chi Medical Foundation. The patients/participants provided their written informed consent to participate in this study.

## Author Contributions

CYS, Y-CC, and Y-KW: study conception and design. C-HL, K-WT, and Y-KW: data collection. P-CH, I-ST, and Y-KW: statistical analysis. P-CH, C-HL, K-WT, and Y-KW: interpretation of results. P-CH and Y-KW: drafting manuscript. CYS and Y-CC: supervision. Y-KW: project administration. All authors reviewed the results and approved the final version of the manuscript.

## Funding

This study was supported by grants from the Taipei Tzu Chi Hospital, Buddhist Tzu Chi Medical Foundation, New Taipei City, Taiwan (TCRD-TPE-111-RT-3 (1/3) and TCRD-TPE-110-44).

## Conflict of Interest

The authors declare that the research was conducted in the absence of any commercial or financial relationships that could be construed as a potential conflict of interest.

## Publisher's Note

All claims expressed in this article are solely those of the authors and do not necessarily represent those of their affiliated organizations, or those of the publisher, the editors and the reviewers. Any product that may be evaluated in this article, or claim that may be made by its manufacturer, is not guaranteed or endorsed by the publisher.
