# Supplementary material for: Efficacy and Safety of Complementary Therapy With Jing Si Herbal Tea in Patients With Mild-To-Moderate COVID-19: A Prospective Cohort Study
Source: Front Nutr. 2022 Mar 14;9:832321. doi: 10.3389/fnut.2022.832321 (PMC8967163; doi:10.3389/fnut.2022.832321)
Supplement: Supplementary file 1 [file Table_1.DOCX]

**Table S1. Medications and HFNC treatments during the study**

| Variable | JSHT group | Control group | *p* value |
| --- | --- | --- | --- |
| Concomitant medications, n (%) |  |  | 0.735 |
| None | 25 (21.4) | 27 (18.9) |  |
| Anti only | 31 (26.5) | 37 (25.9) |  |
| Anti+Dexa | 10 (8.5) | 20 (14.0) |  |
| Anti+Dexa+Rem | 34 (29.1) | 38 (26.6) |  |
| Anti+Dexa+Remd+Toci | 17 (14.5) | 21 (14.7) |  |
| HFNC, n (%) | 10(8.5) | 4(2.8) | 0.077 |
| Anti: antibiotics; Dexa: dexamethasone; HFNC: high-flow nasal cannula; Remd: remdesivir; SpO_2_: Oxyge saturation by pulse oximetry; Toci: tocilizumab; JSHT: Jing Si Herbal Tea | | | |

**Table S2. Key clinical parameters before and after the study**

| Variable | JSHT group | Control group | *p* value |
| --- | --- | --- | --- |
| SpO_2_, % |  |  |  |
| Before (D1) | 95.86 ± 1.86 | 95.96 ± 2.43 | 0.323 |
| After (D8) | 96.82 ± 1.57 | 96.85 ± 1.48 | 0.994 |
| *p* value (D1 and D8) | <0.0001* | 0.002* |  |
| Difference (D8 - D1) | 0.96 ± 2.34 | 0.89 ± 2.99 | 0.556 |
| FiO2, % |  |  |  |
| Before (D1) | 23.28 ± 11.03 | 22.72 ± 9.63 | 0.659 |
| After (D8) | 28.56 ± 19.08 | 27.64 ± 16.70 | 0.408 |
| *p* value (D1 and D8) | 0.004* | <0.0001* |  |
| Difference (D8 - D1) | 5.76 ± 21.02 | 4.92 ± 19.09 | 0.300 |
| Neutrophil to lymphocyte ratio, NLR |  |  |  |
| Before (D1) | 4.52 ± 5.73 | 4.93 ± 4.63 | 0.152 |
| After (D8) | 4.40 ± 6.62 | 4.46 ± 4.65 | 0.133 |
| *p* value (D1 and D8) | 0.142 | 0.008* |  |
| Difference (D8 - D1) | 3.25 ± 6.41 | 3.43 ± 4.49 | 0.259 |
| RT-PCR CT value |  |  |  |
| Before (D1) | 20.75 ± 5.51 | 23.38 ± 6.62 | 0.002* |
| After (D8) | 28.89 ± 5.76 | 28.58 ± 6.47 | 0.611 |
| *p* value (D1 and D8) | <0.0001* | <0.0001* |  |
| Difference (D8 - D1) | 8.14 ± 4.90 | 5.20 ± 6.99 | 0.001* |
| C-reactive protein (CRP), mg/dL |  |  |  |
| Before (D1) | 4.33 ± 4.99 | 3.89 ± 4.33 | 0.601 |
| After (D8) | 0.95 ± 1.63 | 2.14 ± 3.48 | 0.044 |
| *p* value (D1 and D8) | <0.0001* | <0.0001* |  |
| Difference (D8 - D1) | -3.48 ± 5.15 | -2.17 ± 4.96 | 0.044* |
| Brixia score |  |  |  |
| Before (D1) | 2.03 ± 2.22 | 1.27 ± 1.61 | 0.003* |
| After (D8) | 1.52 ± 2.22 | 1.81 ± 2.25 | 0.124 |
| *p* value (D1 and D8) | 0.004* | 0.006* |  |
| Difference (D8 - D1) | -0.50 ± 1.99 | 0.55 ± 2.14 | <0.0001* |

* indicates statistical significance (*p* < 0.05).

**Table S3. Serum Cytokine levels before and after the study**

| Variable | JSHT group | Control group | *p* value |
| --- | --- | --- | --- |
| IL-6, pg/mL |  |  |  |
| Before (D1) | 8.69 ± 17.79 | 11.45 ± 21.78 | 0.549 |
| After (D8) | 7.89 ± 28.12 | 1.74 ± 4.22 | 0.707 |
| *p* value (D1 and D8) | 0.190* | 0.009* |  |
| Difference (D8 - D1) | -0.23 ± 17.85 | -9.71 ± 20.75 | 0.170 |
| IL-8, pg/mL |  |  |  |
| Before (D1) | 9.73 ± 9.43 | 11.83 ± 10.36 | 0.368 |
| After (D8) | 5.67 ± 6.27 | 5.46 ± 4.65 | 0.560 |
| *p* value (D1 and D8) | 0.002* | 0.001* |  |
| Difference (D8 - D1) | -4.06 ± 10.44 | -6.37 ± 9.69 | 0.316 |
| IL-10, pg/mL |  |  |  |
| Before (D1) | 10.10 ± 9.07 | 9.33 ± 7.70 | 0.842 |
| After (D8) | 5.63 ± 5.17 | 5.00 ± 4.10 | 0.643 |
| *p* value (D1 and D8) | <0.001* | 0.003* |  |
| Difference (D8 - D1) | -4.47 ± 10.03 | -4.33 ± 7.17 | 0.766 |
| IL: interleukin. * indicates statistical significance (*p* < 0.05). | | | |

**Table S4. Liver and renal function parameters before and after the study**

| Variable | JSHT group | Control group | *p* value |
| --- | --- | --- | --- |
| AST, U/L |  |  |  |
| Before (D1) | 33.15 ± 20.82 | 37.84 ± 28.10 | 0.142 |
| After (D8) | 24.77 ± 13.83 | 26.29 ± 16.49 | 0.938 |
| *p* value (D1 and D8) | <0.0001* | <0.0001* |  |
| ALT, U/L |  |  |  |
| Before (D1) | 33.78 ± 33.07 | 32.15 ± 31.08 | 0.836 |
| After (D8) | 37.08 ± 32.51 | 40.34 ± 41.28 | 0.871 |
| *p* value (D1 and D8) | 0.043* | 0.023* |  |
| Total bilirubin, mg/dL |  |  |  |
| Before (D1) | 0.57 ± 0.21 | 0.62 ± 0.41 | 0.907 |
| After (D8) | 0.67 ± 0.33 | 0.63 ± 0.33 | 0.219 |
| *p* value (D1 and D8) | 0.003* | 0.030* |  |
| BUN, mg/dL |  |  |  |
| Before (D1) | 15.52 ± 22.06 | 14.90 ± 10.62 | 0.570 |
| After (D8) | 14.26 ± 8.37 | 15.05 ± 12.97 | 0.784 |
| *p* value (D1 and D8) | 0.467 | 0.051 |  |
| Creatinine, mg/dL |  |  |  |
| Before (D1) | 0.83 ± 0.41 | 1.14 ± 2.08 | 0.093 |
| After (D8) | 0.71 ± 0.27 | 0.97 ± 2.26 | 0.375 |
| *p* value (D1 and D8) | <0.0001* | <0.0001* |  |
| AST: aspartate aminotransferase; ALT: alanine aminotransferase; BUN: blood urea nitrogen; JSHT: Jing Si Herbal Tea. * indicates statistical significance (*p* < 0.05). | | | |
